# An Unusual Case of Adult-Onset Still's Disease with Hemophagocytic Syndrome, Necrotic Leukoencephalopathy and Disseminated Intravascular Coagulation

**DOI:** 10.1155/2014/128623

**Published:** 2014-03-04

**Authors:** Rajaie Namas, Naveen Nannapaneni, Malini Venkatram, Gulcin Altinok, Miriam Levine, J. Patricia Dhar

**Affiliations:** ^1^Department of Internal Medicine, Division of Rheumatology, Wayne State University, Detroit, MI 48201, USA; ^2^Department of Internal Medicine, Wayne State University, Detroit, MI 48201, USA; ^3^Department of Radiology, Wayne State University, Detroit, MI 48201, USA

## Abstract

*Case*. A 34-year-old African-American female with a history of adult-onset Still's disease presented to an outside hospital with oligoarthritis. She experienced a generalized tonic-clonic seizure *en route* via ambulance, was intubated upon arrival, and transferred to the intensive care unit for treatment of suspected pneumonia and sepsis. She subsequently developed generalized cutaneous desquamation that progressed despite the cessation of antibiotics and other potential offending drugs which required transfer to our hospital's burn unit. She was suspected to have reactive hemophagocytic syndrome based on her clinical presentation of fever, rash, polyarthritis, elevated liver enzymes, coagulopathy, splenomegaly, normocytic anemia, thrombocytopenia, hypertriglyceridemia, hyperferritinemia, and hemophagocytosis visualized in bone marrow biopsy specimen. Magnetic resonance imaging demonstrated necrotic demyelination of the deep white matter and corona radiata. The patient developed multiorgan dysfunction and DIC without any other attributable etiology. Despite aggressive broad spectrum therapy and high dose of steroids she progressively deteriorated and eventually expired. *Conclusion*. Previous publications have highlighted the prevalence of necrotic leukoencephalopathy in children with familial hemophagocytic syndrome. Our patient demonstrated some uncommon features complicating her HLH including DIC and necrotic leukoencephalopathy, which are very rare entities in AOSD.

## 1. Introduction

Still's disease was first described by George Still in 1896. Adult-onset Still's disease (AOSD) describes a similar condition which affects adults and is generally constituted by recurrent high grade fever with concomitant salmon-colored rash of the trunk and extremities, oligo- and later polyarthritis, pharyngitis, splenomegaly, lymphadenopathy, and less commonly pericarditis or pleural effusions [1]. Laboratory evaluation typically demonstrates elevated erythrocyte sedimentation rate, leukocytosis, and elevated liver enzymes [2].

Hemophagocytic syndrome (HS), also termed hemophagocytic lymphohistiocytosis (HLH), is a syndrome of immune activation manifested by signs and symptoms of inflammation [3]. HS is diagnosed based on the fulfillment of 5/8 criteria as in Table 1 [4]. Histopathological evaluation demonstrates lymphocytes, macrophages and hemophagocytosis in bone marrow, spleen, cerebral spinal fluid, and liver [4]. Other common clinical manifestations include central nervous system involvement, coagulopathy, liver dysfunction, jaundice, edema, and skin rashes [4]. A variety of CNS manifestations of HS including seizures, ataxia, and meningitis have been reported [5, 6]. HS can be familial as in young children or reactive as in patients with underlying infection, lymphoma, organ transplant, systemic lupus erythematosus, or AOSD [7–9].

## 2. Case Report

**Figure 1:**
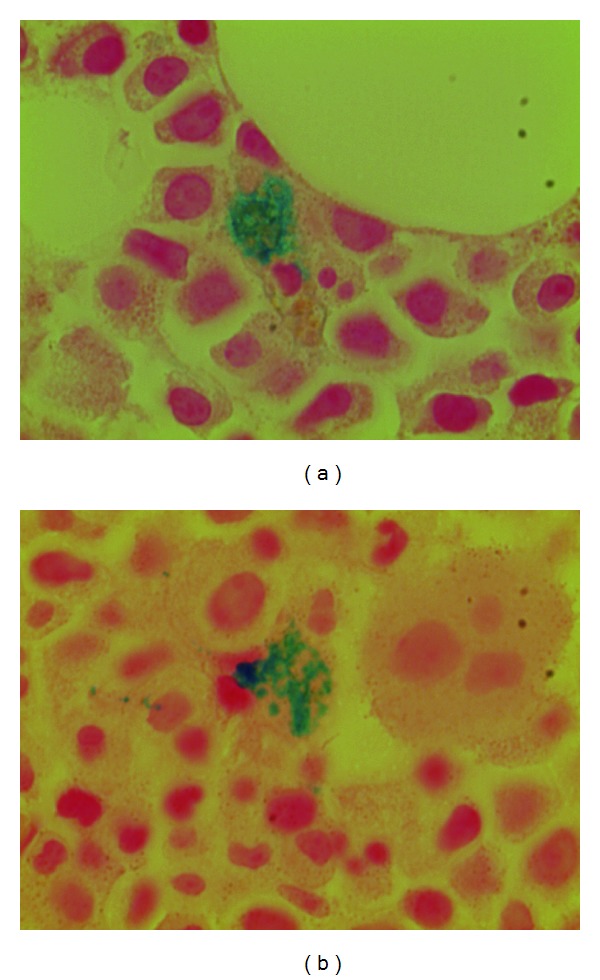
Bone marrow biopsy demonstrating hemophagocytosis.

A 34-year-old African-American female was diagnosed with adult-onset Still's disease (AOSD) 3 months prior to this admission based on episodic fever of unknown origin, gastrointestinal manifestations of episodic diarrhea, vomiting, leukocytosis, oligoarthritis, nonspecific facial rash, weight loss, and elevated ferritin level. Extensive infectious workup was unremarkable. Hematological work up was unremarkable except for leukocytosis, generalized lymphadenopathy and hepatosplenomegaly. A lymph node biopsy demonstrated reactive lymphadenitis and was unremarkable for any hematological malignancy or infection. Upper endoscopy and colonoscopy, including ileal and colon biopsies, and abdominal imaging with CT scan were unremarkable for a primary gastrointestinal disease. Synovial fluid analysis demonstrated an inflammatory fluid pattern. Extensive infectious evaluation with imaging, cultures (sputum, blood, urine, and cervical and all the other potential sources), and serologies for atypical infections including viral and fungal diseases failed to demonstrate an infectious etiology. Synovial cultures were unremarkable for infections including Whipple's disease (*Tropheryma whipplei*). Other autoimmune workups for connective tissue disease and vasculitides were unremarkable. She was eventually diagnosed with Still's disease that was supported by high ferritin levels of 7000 ng/mL which increased to 22000 ng/mL (18–160 ng/mL). Bone marrow biopsy demonstrated mild hemophagocytosis (Figure 1). However, patient did not demonstrate pancytopenia or other features of HS. She responded significantly and rapidly to a trial of prednisone 40 mg daily. Corticosteroid sparing agent such as methotrexate or Kineret (Anakinra) was suggested; however it was unaffordable due to financial constraints and social situation. Attempts to wean her steroid dose under 30 mgs resulted in recurrences many times.

Four days prior to this admission she noted worsening of her oligoarthritis and was evaluated in a local emergency department. She reported compliance with her medication regimen of prednisone 40 mg orally daily. She was discharged on pain medications and antibiotics for a possible urinary tract infection. The next day she was evaluated at another hospital for worsened arthralgia and mental status changes. Her hospital course was further complicated by worsening of her mental status requiring intubation and eventually shock. She was suspected to have pneumonia and was treated with antibiotics and vasopressors for her shock. Cultures were negative for any growth. A computed tomography (CT) of the head was unremarkable. She was extubated after clinical improvement. However, 5 days later she was noted to develop desquamation of large patches of the skin on her chest, extremities, and abdomen. At this point, potential offending drugs including antibiotics were discontinued due to lack of evidence to support an infectious process and she was transferred to our facility's burn unit with a concern for Stevens-Johnson Syndrome/Toxic Epidermic Necrolysis.

**Figure 2:**
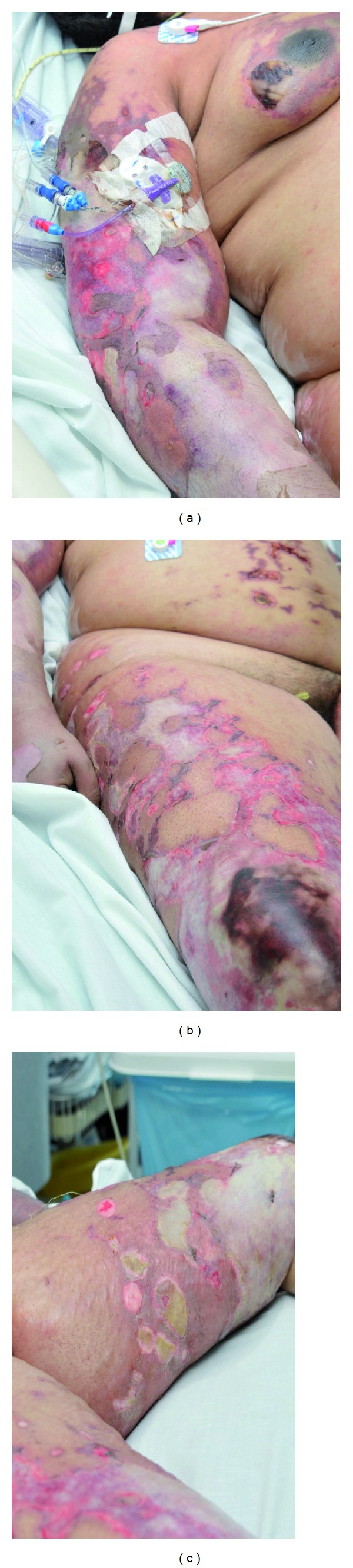
Large patches of denuded skin over the chest, breasts, arms, legs, upper back, and buttocks with nondenuded areas appearing mottled and ecchymotic.

**Figure 3:**
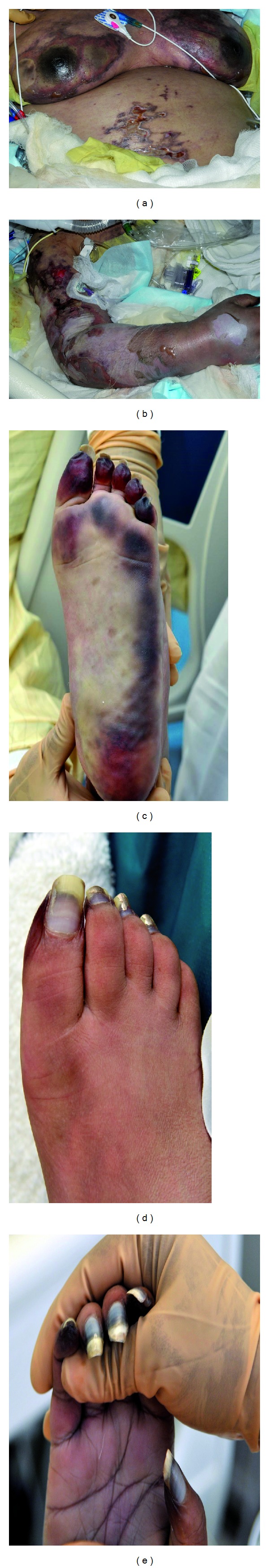
Dry gangrenous changes in the fingertips and toes.

On arrival, she was in moderate distress and found to be stuporous. She was normotensive, tachycardiac, tachypneic, and febrile. Her BMI was 40.7 kg/m^2^. She was diffusely edematous, with large patches of denuded skin over the chest, breasts, arms, legs, upper back, and buttocks with nondenuded areas appearing mottled and ecchymotic (Figure 2). There were no mucosal abnormalities. Cardiopulmonary exam revealed tachycardia and diminished breath sounds. Abdominal examination revealed mild splenomegaly with soft consistency. Extremities were cold to palpation without palpable pulses. All fingertips and toes were shriveled and black with dry gangrenous changes (Figure 3). Neurological exam was limited due to mental status changes, but no localizing signs or focal neurological deficit could be identified. The rest of physical exam was unremarkable.

Preliminary labs demonstrated leukocytosis, normocytic anemia, thrombocytopenia, transaminitis, marked elevation of the erythrocyte sedimentation rate, and C-reactive protein and, notably, ferritin levels were profoundly elevated 25,512 ng/mL (normal 18–200). Urine analysis demonstrated moderate hematuria and proteinuria. Urine sediment demonstrated muddy brown casts consistent with acute tubular necrosis. Rheumatoid factor, anti-cyclic citrullinated peptide antibodies (anti-CCP), Extractable Nuclear Antigens (ENA), C and P-ANCA (anti-neutrophil cytoplasmic antibodies), serum protein electrophoresis (SPEP), and urine protein electrophoresis (UPEP) levels were normal. Infectious workup including Epstein-Barr virus (EBV), human immunodeficiency virus (HIV), cytomegalovirus (CMV), rapid plasma reagin (RPR), respiratory syncytial virus (RSV), influenza PCR, hepatitis A, hepatitis B, hepatitis C, Varicella, human T-lymphotropic virus type I and II, herpes simplex virus 1 and 2, human herpesviruses (HHV6, 7, and 8), adenovirus, aspergillus, parvovirus, and Bartonella were all negative. Cultures of the blood, CSF, urine, fungal, mycobacterium, bronchial washings, cervix and rectum, and any other possible source of infection were all negative. Neuron specific enolase level was 13.5 and Interleukin 2 (soluble CD-25) level of 1929 pg/mL (normal 0–1033). Familial HLH and PRF1 gene sequencing was negative.

A chest radiograph demonstrated cardiomegaly with mild pulmonary vascular congestion. Echocardiogram evaluation exhibited mildly decreased left ventricular systolic function with hypokinesis of the inferior and inferolateral walls of the left ventricle. No valvular vegetation was reported. CT of the abdomen revealed splenomegaly with heterogeneous enhancement and areas of hypoattenuation/enhancement along the splenic periphery and mild lymphadenopathy primarily in the upper abdomen and splenic hilum. High-resolution CT of the thorax demonstrated patchy airspace opacities in the upper lobes and lower lobes bilaterally, suggestive of acute respiratory distress syndrome.

**Figure 4:**
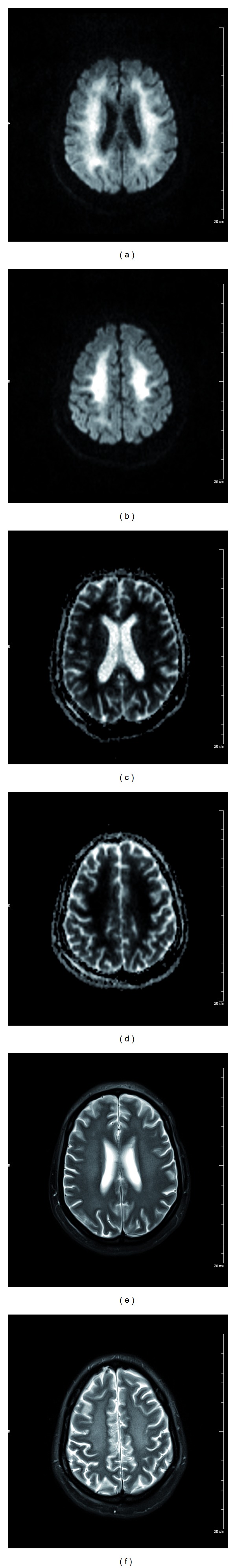
(a) DWI, Diffusion weighted images bilateral and symmetric diffusion restriction in the deep white matter. (b) DWI, Diffusion weighted images show bilateral and symmetric diffusion restriction in the corona radiata. (c) ADC map shows loss of the increased signal seen in the DWI images in the bilateral deep white matter. (d) ADC map shows loss of the increased signal seen in the DWI images in the bilateral corona radiata. (e) Axial T2 TSE FS (Blade) acquired in 4 mm thickness, shows hyperintense T2 signal abnormality in the deep white matter. (f) Axial T2 TSE FS (Blade) acquired in 4 mm thickness, shows hyperintense T2 signal abnormality in the corona radiata.

Magnetic resonance imaging (MRI) of the brain showed an extensive bilateral and symmetric hyperintense T2 signal abnormality and diffusion restriction throughout the deep white matter and corona radiata, sparing the subcortical U fibers without evidence of enhancing lesions, findings which were reported as consistent with necrotic leukoencephalopathy (Figures 4(a)–4(f)). An electroencephalogram was performed revealing diffuse slowing consistent with a moderately severe diffuse encephalopathy.

Bone marrow biopsy demonstrated hemophagocytosis as shown in Figure 1. A skin biopsy performed at the outside hospital demonstrated vascular congestion with mild dermal edema, mild perivascular lymphocytic infiltrate, and rare mast cells.

Patient was started on high dose pulse intravenous steroids. Hematology recommended an aggressive immunosuppressive regimen including intravenous dexamethasone, etoposide, and intrathecal methotrexate for possible HLH after consultation with additional HLH experts across the country. After initiation of high dose steroids but prior to initiation of etoposide or methotrexate the patient deteriorated, requiring reintubation for acute respiratory distress and vasopressors for shock. She further developed anuric renal failure, lactic acidosis, coagulopathy, and nonsustained ventricular tachycardia requiring an amiodarone drip. At this point, the family decided to avoid further aggressive care and withdraw life support. The patient eventually expired after a progressive downhill course totaling 8 days at our institution.

Postmortem examination was conducted with informed consent from the family and revealed interstitial inflammation in the heart, lungs, gastrointestinal, urinary bladder, and skin. Patchy interstitial and intramuscular fibrosis with multiple vegetation with superimposed organized thrombi in the heart. Cultures of the vegetation were negative. Several microfibrin thrombi were identified in the lumens of small- and medium-sized blood vessels in the lungs. Brain biopsy revealed extensive Alzheimer type 2 cells with microvascular changes due to reactive inflammatory disease. No infection or tumor was identified in any of the organs.

## 3. Discussion

Hemophagocytic lymphohistiocytosis (HLH) is a hyperinflammatory syndrome with high mortality [10, 11]. HLH is characterized by acute fever, hepatosplenomegaly, lymphadenopathy, pancytopenia, and raised levels of serum ferritin, triglycerides, and liver enzymes.

Our patient with a recent diagnosis of Still's disease presented with polyarthritis, fever, and encephalopathy with altered mentation, seizures, ischemia resulting in dry gangrene in all extremities, skin desquamation, lymphadenopathy, and splenomegaly. Workup demonstrated anemia, leukocytosis, thrombocytopenia, coagulopathy, elevated inflammatory markers, high ferritin level of 25,000, high triglyceride, and elevated liver enzymes. Imaging demonstrated patchy pulmonary infiltrates suggestive of acute respiratory distress syndrome in the clinical setting of multiorgan failure. Echocardiographic evaluation exhibited mildly decreased left ventricular systolic function with no valvular vegetation reported. Bone marrow smear and biopsy demonstrated hypercellular bone marrow of 80% cellularity with greatly increased myeloid leukemogenesis and decreased erythrocytogenesis without any cellular dysplasia noted and normal appearing of small plasma cells. Hemophagocytosis in marrow was noted. An extensive infectious workup was unremarkable for any infectious etiology as a possible explanation of her multiorgan involvement. The diagnosis of HS complicating AOSD in our patient was a result of several clinical findings and laboratory parameters which fulfilled required diagnostic criteria in the absence of an infectious or neoplastic cause for her recent deterioration.

In addition, our patient demonstrated many atypical features aside from HLH complicating AOSD. This includes DIC and necrotizing encephalopathy on imaging which is rarely reported, especially in adults. This was followed by shock and progressive multisystem organ failure. No case has been reported to our knowledge with these two complications occurring concomitantly in an adult patient as a complication of HLH. Further, even prior to this admission our patient manifested with features of GI symptoms of episodic diarrhea every time she had a disease flare reflecting possible GI inflammation which is atypical. She terminally developed multiorgan failure complicating her HLH as supported by autopsy.

Our patient developed HS with months of manifesting with the features of AOSD. Literature review suggested that HS can occur at any time when there is established diagnosis of AOSD. Flare of AOSD and HS are clinically indistinguishable, except for a high frequency of pleuritis and acute respiratory distress syndrome. Laboratory diagnosis may be helpful in making a diagnosis of HS during a flare of AOSD. Leukopenia or thrombocytopenia is uncommon in AOSD. High ferritin level is generally more typical of AOSD. Nevertheless a very high serum ferritin concentration (>10 000 mg/L) is more suggestive of HS complicating AOSD [12]. Raised serum triglyceride level is considered to be a good marker of HS and may be useful in its diagnosis [13] but it has not been specifically analyzed in flare of AOSD. In our patient, serum triglycerides were markedly increased during the diagnosis of HS.

Disseminated intravascular coagulation is a serious complication shared by both HS and AOSD and is associated with high mortality [14–16]. Although it is described more in AOSD case reports, J-B Arlet et al. observed disseminated intravascular coagulation in one third of the patients with AOSD-related HS and was associated with severe liver cytolysis and high mortality.

Our patient developed ischemia and coagulopathy with increased levels of fibrin degradation products, prolongation of prothrombin time and activated partial thromboplastin time, and a low fibrinogen level overall suggesting a diagnosis of DIC in a terminally ill patient with multiorgan dysfunction. Vascular surgery did not suggest the use of vasopressors as the cause of the ischemia. This was further supported by autopsy which demonstrated thrombosis in the lumen of the blood vessels. Although mortality is low in patients with AOSD, most deaths are due to DIC or ARDS, when the diagnosis is associated with HS [2, 17].

Recurrent gastrointestinal symptoms manifesting as a part of clinical flare was another atypical feature of our patient before developing HLH. An extensive workup for primary gastrointestinal diseases contributing to her symptoms such as Whipple's disease and celiac disease was unremarkable. Multiple endoscopic biopsies with cultures failed to demonstrate anything outside a nonspecific inflammation. Recurrent GI inflammation manifesting as a part of clinical flare is unusual. Predominant GI symptoms are atypical in AOSD but Meeths et al. reported 5 patients who presented with abdominal pain and chronic diarrhea and concluded that severe gastrointestinal symptoms may present before typical clinical manifestations of HLH. This unusual manifestation can add to the diagnostic dilemma and needs to be considered in patients with AOSD. These symptoms likely occurred from the inflammation of the stomach and small intestine. This was previously demonstrated by Zhao et al. by interstitial inflammation in the mucosa of the alimentary canal in postmortem examination in a patient with AOSD [18, 19].

While previous publications have highlighted the prevalence of necrotic leukoencephalopathy in children with familial hemophagocytic syndrome, our adult patient presented with necrotizing leukoencephalopathy in the context of DIC complicating her reactive hemophagocytic syndrome, a far less common presentation [5]. Several case reports indicate that children with necrotic leukoencephalopathy in the setting of hemophagocytic syndrome demonstrated recovery of central nervous system dysfunction [5]; however there is no study evaluating outcomes in adults.

Bone marrow biopsy, often performed to eliminate infection or neoplasm, is less sensitive than bone marrow aspirate. In a study by J-B Arlet et al. bone marrow biopsy was considered to be normal in two thirds of patients, whereas HS was demonstrated in the bone marrow aspirate. This was also noted by others who report positive bone marrow aspirate and negative bone marrow biopsy for HS [13, 20]. Autopsy results revealed interstitial inflammation in the heart, lungs, gastrointestinal, urinary bladder, and skin. Patchy interstitial and intramuscular fibrosis with multiple vegetation with superimposed organized thrombi in the heart. Cultures of the vegetation were negative. Several microfibrin thrombi were identified in the lumens of small- and medium-sized blood vessels in the lungs. Brain biopsy revealed extensive Alzheimer type 2 cells with microvascular changes due to reactive inflammatory disease. No infection or tumor was identified in any of the organs.

To summarize, our patient presented with new onset adult Still's disease manifested with fever, altered mentation, seizures, ischemia resulting in dry gangrene in all extremities, cutaneous denudation, and splenomegaly. Workup demonstrated anemia, thrombocytopenia, leukocytosis, high ferritin, and high triglyceride level with bone marrow changes suggestive of HLH. Course was complicated with DIC considered given the ischemia, coagulopathy, and multiorgan involvement in the absence of any other valid explanation. Brain imaging revealed hyperintense T2 findings and diffusion restriction in the deep white matter bilaterally, consistent with necrotizing leukoencephalopathy. Extensive infectious workup was unremarkable for a possible infectious etiology.

This case underscores the need to be vigilant for these atypical manifestations in HLH including demyelination within the central nervous system, which is a more reported complication in children with familial HLH rather than adults.

## Figures and Tables

**Table 1:** Diagnostic criteria of hemophagocytic syndrome.

(1) Fever	
(2) Spleenomegaly	
(3) Cytopenia in 2 of 3 cell lines	
(4) Hypertriglyceridemia	
(5) Hemophagocytosis in either bone marrow, lymph nodes or the spleen	
(6) Low/absent NK cell activity	
(7) Hyperferritinemia (>500 *μ*g/L)	
(8) High levels of soluble IL-2	

Courtesy from Henter et al. [4].

## Abbreviations

AOSD: Adult-onset Still's disease
HS: Hemophagocytic syndrome
HLH: Hemophagocytic lymphohistiocytosis
GI: Gastrointestinal
CT: Computed tomography
ENA: Extractable nuclear antigens
ANCA: Antineutrophil cytoplasmic antibodies
DIC: Disseminated intravascular coagulation.
